# Lightweight CNN and transformer detectors for real-time single-fruit date-variety recognition on edge GPU hardware

**DOI:** 10.1371/journal.pone.0349608

**Published:** 2026-10-27

**Authors:** Adnan Miski, Moayad Alshangiti

**Affiliations:** 1 Department of Industrial Engineering, Faculty of Engineering-Rabigh, King Abdulaziz University, Jeddah, Saudi Arabia; 2 Department of Software Engineering, College of Computer Science and Engineering, University of Jeddah, Jeddah, Saudi Arabia; Hohai University, CHINA

## Abstract

Manual sorting of date fruits is time-consuming and labor-intensive, while automated optical sorting lines remain costly for small- and medium-sized enterprises (SMEs). This study presents a deployment-oriented benchmark of five lightweight object detectors for single-fruit date-variety recognition, namely the Convolutional Neural Network (CNN)-based YOLOv8, YOLOv10, YOLOv11, and YOLOv12 Nano variants and the Transformer-based D-Fine-Nano, with RT-DETR-Large included as a high-capacity accuracy reference. Models were evaluated on 1,658 controlled-environment images of nine commercial varieties using grouped 5-fold cross-validation, which kept identified repeat photographs of the same fruit within the same cross-validation fold. Latency was measured on an NVIDIA T4-class edge GPU/industrial-PC accelerator, and the CNN detectors were also exported to Open Neural Network Exchange (ONNX) and TensorRT half-precision (FP16) engines. All Nano models met the 30 frames-per-second threshold for model-side detection latency under the tested conditions and achieved high accuracy, while a Friedman–Nemenyi analysis detected no statistically significant accuracy difference among the lightweight models. Under TensorRT FP16, YOLOv10-Nano achieved the lowest model-side end-to-end latency of 6.27 ms with no observed accuracy degradation. YOLOv8-Nano attained the lowest inference-stage latency of 3.31 ms, but its mean average precision (mAP@50–95) decreased by 1.2 percentage points after TensorRT FP16 conversion. YOLOv12-Nano had the numerically highest mean cross-validation mAP@50–95 and the lowest fold-to-fold variability, albeit at higher latency. These findings characterize model-level detector behavior rather than a complete sorting line. Within that scope, YOLOv10-Nano represents one practical configuration for a T4/TensorRT FP16 pipeline, while the other models offer different speed, stability, and deployment trade-offs, providing SMEs with a well-characterized starting point for the vision component of affordable date-sorting systems.

## Introduction

The date fruit industry presents a particularly suitable context for automated visual inspection because of the scale of its postharvest sorting challenge. Approximately 100 million date palms are cultivated worldwide, with the majority located in the Middle East [1]. Global production increased from 3.4 million metric tons in 1990 to 9.6 million metric tons in 2021; that year, Egypt and Saudi Arabia produced roughly 1.7 and 1.5 million metric tons of dates, representing 18% and 16% of world output, respectively [2]. Dates are nutritionally dense, containing 40–80% sugars and appreciable amounts of dietary fiber, minerals, and antioxidants [3]. In food-insecure regions, they also serve as a source of energy and micronutrients [3].

Despite this economic and nutritional importance, postharvest handling remains a significant bottleneck. Dates of different maturity stages should not be packaged together because they can have adverse mutual effects and market preferences differ; however, traditional sorting by maturity is time-consuming, costly, and tedious [4]. In Algeria alone, for instance, there are more than 17 million date palms and over 800 recognized varieties [5], yet manual sorting persists. Producers face practical challenges in harvesting and packaging: workers often shake palm bunches so ripe fruits fall into containers and then manually separate immature and damaged fruits, leading to long working hours and errors associated with repetitive tasks [6]. Similar issues occur in other horticultural packing lines; in sweetpotato packinghouses, for example, manual defect sorting accounts for up to 50% of total labor, while sorting quality is affected by subjectivity, fatigue, and musculoskeletal strain [7].

While high-throughput optical sorting machines exist, they often remain prohibitively expensive for small- and medium-sized enterprises (SMEs) [7]. For the vision component of more affordable sorting systems, models must combine high recognition accuracy with low inference latency on cost-effective hardware. Computer vision based on affordable graphics processing unit (GPU) accelerators has therefore become an active research area, reflecting the broader adoption of deep learning (DL) in agriculture [8]. A recent systematic review of 56 studies on fruit inspection found that RGB cameras and DL classifiers such as ResNet and VGG dominate current practice [9]. A focused review of Convolutional Neural Network (CNN) models for external fruit quality inspection further confirmed that transfer learning with pre-trained architectures is the dominant approach [10], and Olorunfemi et al. highlighted the increasing adoption of DL for fruit sorting and grading while noting persistent challenges in system robustness and scalability [11].

In the domain of date fruit classification, various CNN architectures have been evaluated. Nasiri et al. trained a VGG-16-based model to distinguish healthy from defective dates and to predict ripening stages, achieving an overall accuracy of 96.98% [4], and Altaheri et al. classified date-fruit type and maturity for robotic harvesting using AlexNet and VGG-16 [12]. Abden et al. constructed an ensemble of MobileNetV2, EfficientNet, and DenseNet201 to achieve 99.93% classification accuracy across 20 Algerian date varieties [5]. Rybacki et al. developed a CNN-based classification model for Algerian date varieties using color and geometric features, achieving high classification accuracy [13]. Alresheedi et al. explored feature fusion for nine date varieties, noting the difficulty of distinguishing among more than 200 known cultivars [14], and Pérez-Pérez et al. evaluated eight CNN architectures, including VGG, ResNet, and Inception variants, for sorting ripe Medjool dates [6]. Detection-based approaches have also emerged. Almutairi et al. compared three You Only Look Once (YOLO) detectors, YOLOv5, YOLOv7, and YOLOv8, for detecting nine date varieties in 1,735 images [15], and more recently, Hassan et al. combined DenseNet121 with a Squeeze-and-Excitation attention block for date fruit classification, achieving 98.25% accuracy compared with 96.04% for YOLOv8n [16].

However, many reported fruit-inspection systems have been evaluated under laboratory or static conditions rather than under the real-time constraints of an operating sorting line [9]. Modern conveyor-belt sorting systems require detection models that can operate at or above 30 frames per second (FPS) on affordable GPU accelerators while maintaining high accuracy [17]. Some studies have demonstrated inline deployment; for example, Fan et al. deployed a CNN-based vision system for online detection of defective apples on a commercial sorting machine [18], and Zhu and Spachos combined edge-side support vector machines with cloud-based YOLO models for food grading [19]. For dates, YOLOv5 has been explored for postharvest surface defect detection [20]. Although recent Nano-scale CNN and Transformer detectors report competitive accuracy on public benchmarks, their inference behavior on cost-effective GPU accelerators is less frequently characterized under deployment-oriented conditions. Deployment-oriented benchmarks comparing recent Nano-scale detectors across multiple YOLO generations for date sorting also remain scarce. The emergence of Transformer-based detectors, such as RT-DETR and D-Fine, further raises the question of whether these architectures offer accuracy advantages over CNN-based detectors within the latency budgets of industrial sorting lines.

This study addresses these gaps through a deployment-oriented comparative benchmark of lightweight object detectors for single-fruit date-variety recognition. The primary contribution is the controlled and reproducible benchmarking and deployment characterization of existing Nano-scale CNN and Transformer detectors, rather than a new detector architecture or a validated date-sorting system. Specifically, we characterize detection accuracy and statistical stability, compare model-side inference latency on A100 and T4 GPU hardware, and evaluate Open Neural Network Exchange (ONNX) and TensorRT half-precision (FP16) deployment behavior for the YOLO CNN models on the T4. In this study, “edge” refers to tethered industrial-PC GPU acceleration, including T4-class accelerators, rather than to low-power embedded modules such as the NVIDIA Jetson family. We evaluate the Nano variants of the YOLO family of models (v8, v10, v11, and v12) alongside the Transformer-based D-Fine-Nano, with the high-capacity RT-DETR-Large included as a fixed accuracy reference rather than a lightweight competitor. The evaluation uses a public controlled-environment date fruit dataset spanning nine commercial varieties, whose uniform illumination and clean background approximate the singulated imaging conditions of illuminated conveyor-belt inspection while remaining a single-object setting. Performance is quantified through mean average precision (mAP), precision, recall, floating-point operations (FLOPs), model size, and inference latency. By identifying models that balance high accuracy with strict operational constraints, this work aims to inform—rather than validate—the development of affordable, automated postharvest sorting frameworks for the date palm industry.

## Materials and methods

This section describes the experimental design, dataset, augmentation, model architectures, computing environment, training configuration, evaluation metrics, and model export used to benchmark lightweight detectors for single-fruit date-variety recognition.

### Experimental design

The experiments were conducted in two distinct phases, as illustrated in Fig 1, to ensure both comprehensive hardware profiling and statistical reliability.

- *Phase 1, Single-Run Benchmark:* The primary objective of this phase was to establish baseline metrics for accuracy, inference speed, and hardware efficiency. Models were trained and evaluated using a fixed 80/10/10 split, with the training subset expanded using offline augmentation.
- *Phase 2, Grouped 5-Fold Cross-Validation:* To reduce dependence on a single data split and assess model stability, grouped 5-fold cross-validation was performed [21]. Identified repeat photographs of the same physical fruit were constrained to remain within the same fold, and offline augmentation was replaced with each framework’s online augmentation.

**Fig 1:**
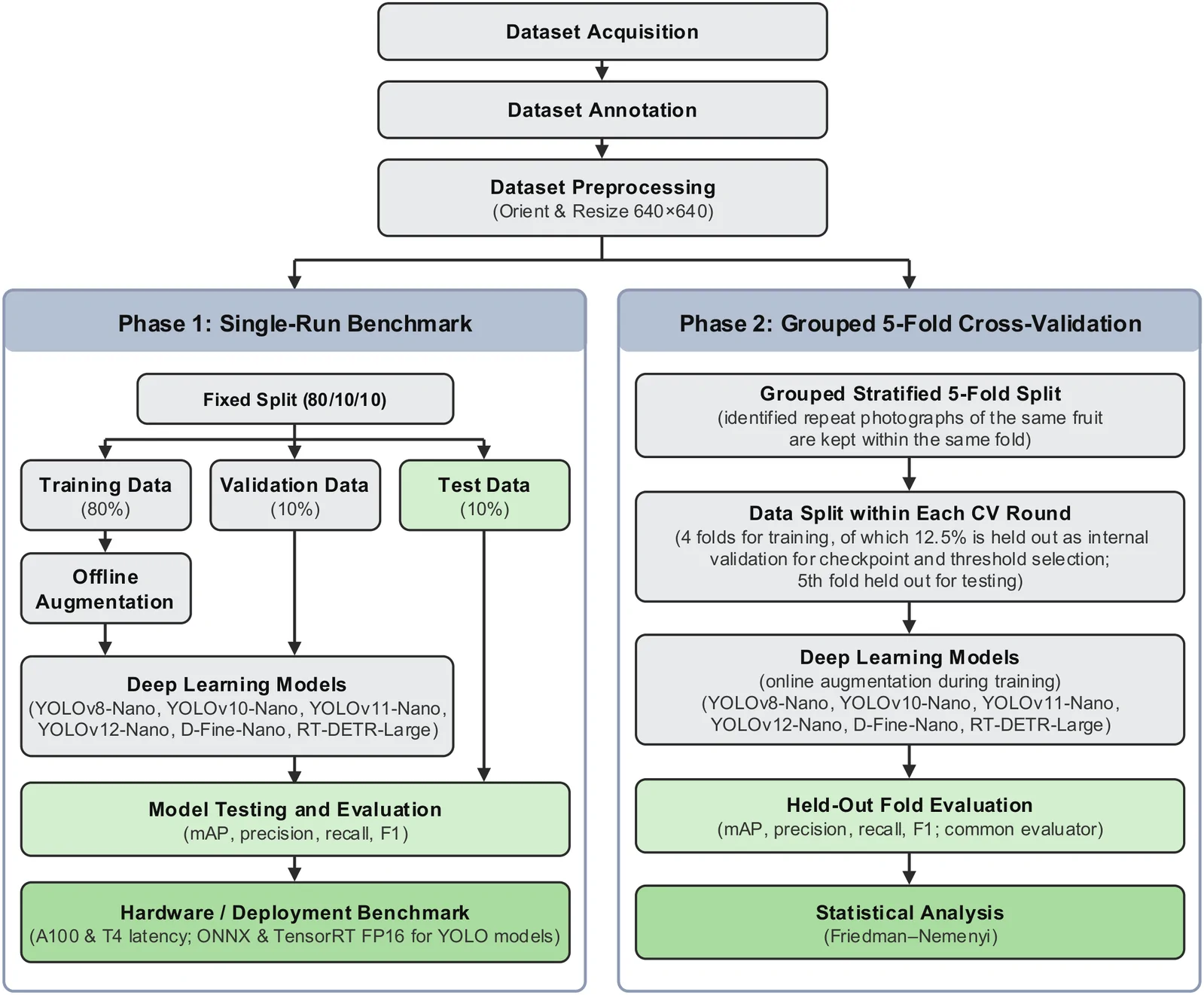
Model-level benchmarking workflow. The diagram illustrates the experimental methodology, including data acquisition, manual annotation, augmentation, model training, testing, and evaluation.

### Dataset acquisition and partitioning

The study utilizes the “Date Fruit Image Dataset in Controlled Environment” [22], which we adapted for object detection. The dataset contains 1,658 high-resolution images distributed across nine commercial date varieties in Saudi Arabia, comprising 264 Sokari, 232 Meneifi, 190 Galaxy, 177 Nabtat-Ali, 175 Ajwa, 171 Shaishe, 168 Sugaey, 146 Rutab, and 135 Medjool images. While previous works have utilized this dataset for basic image classification tasks [23], our study adapts it for object detection to benchmark bounding-box inference under a controlled single-fruit setting relevant to conveyor-belt inspection. Fig 2 presents representative samples of these varieties. The controlled capture environment, featuring uniform illumination and a clean background, approximates simplified singulated imaging conditions that may occur in illuminated conveyor inspection, while remaining substantially less complex than an operational sorting line.

**Fig 2:**
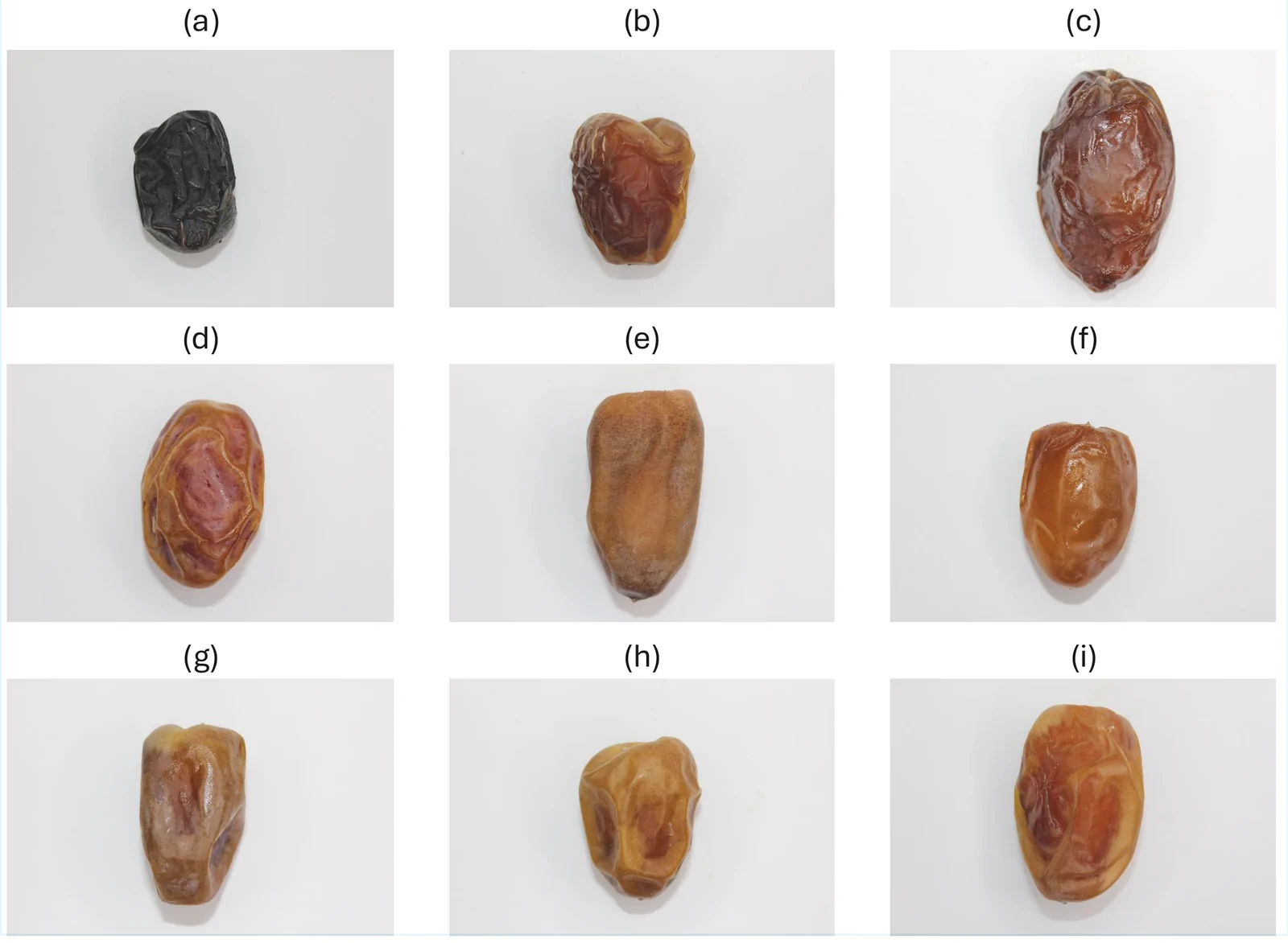
Representative images from the dataset. Examples of each variety captured under consistent lighting conditions. The dataset classes are a) Ajwa, b) Galaxy, c) Medjool, d) Meneifi, e) Nabtat-Ali, f) Rutab, g) Shaishe, h) Sokari, i) Sugaey.

Captured in a controlled setting, the images possess an average resolution of 7.96 MP (median dimensions of 3456×2304 pixels), providing rich textural details necessary for distinguishing subtle varietal features. The dataset was manually annotated using the Roboflow platform [24]. Because each image contains a single, centered fruit, it carries exactly one bounding-box annotation, as shown in Fig 3.

**Fig 3:**
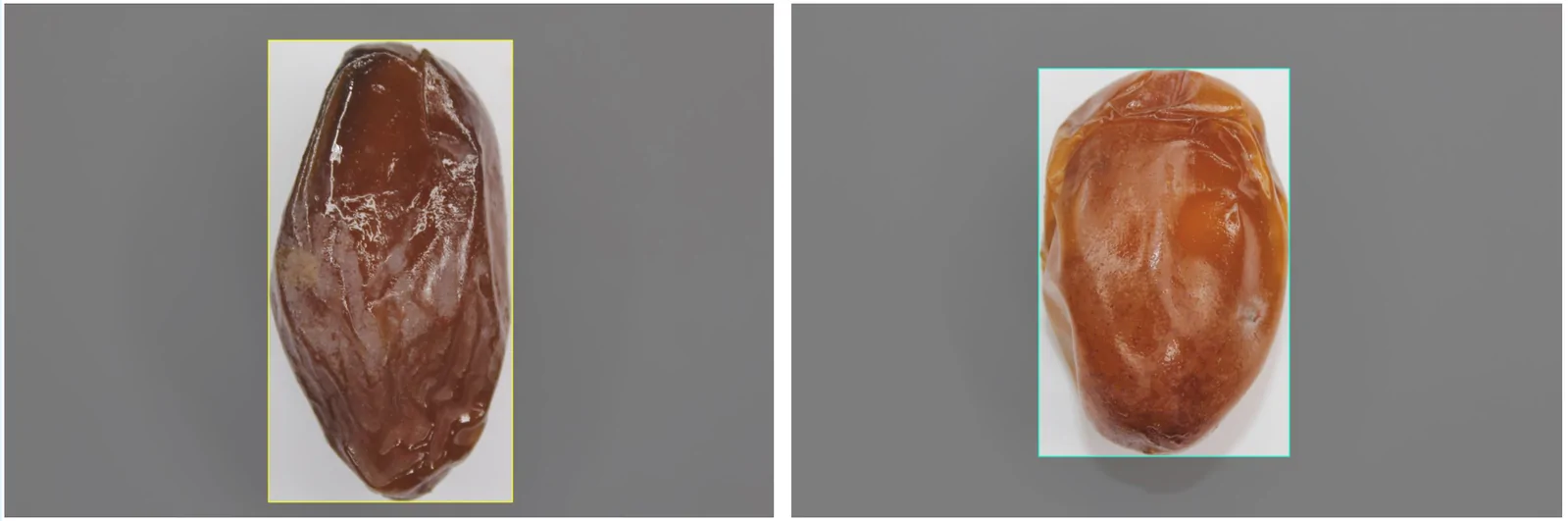
Sample images. Examples from the dataset showing the manual bounding-box annotations.

We deliberately frame the task as object detection rather than image classification for two reasons. First, it yields a single, unified pipeline—localization and variety identification in one forward pass—that extends directly to the multi-fruit, non-centered scenes expected on a live conveyor, whereas a pure classifier would need to be paired with a separate detector or singulation stage before deployment. Second, it allows us to benchmark the detection architectures (YOLO, D-Fine, RT-DETR) that are the realistic candidates for such systems. We nonetheless emphasize that, on this single-object dataset, the localization sub-task is comparatively easy: each image contains one large, centered object against a clean background. The present results are therefore best interpreted as detector behavior under near-classification conditions, and not as evidence of multi-object detection capability.

The source dataset does not provide per-image provenance metadata such as capture batch, palm or tree of origin, or harvest session. An image-similarity audit was therefore performed using cosine similarity between 512-dimensional features extracted from the annotated fruit crops with an ImageNet-pretrained ResNet-18 [25]. Across the 1,658 images, no pair exceeded a similarity of 0.99 (maximum 0.9898). All 148 same-variety pairs with a similarity of at least 0.95 were inspected visually, and 17 pairs, involving 34 images (2.1% of the dataset), were identified as repeat photographs of the same physical fruit; no image belonged to more than one pair. These pairs were constrained to remain within the same cross-validation fold (see Cross-validation protocol).

Fig 4 and Table 1 detail the class distribution. The classes are moderately imbalanced, with 135–264 images per variety, a ratio of roughly 2:1 between the largest and smallest class. For Phase 1, the data were randomly partitioned into training (80%), validation (10%), and testing (10%) subsets, which approximately preserved each variety’s proportion; the Phase 2 folds were stratified by variety as described in the Cross-validation protocol section. To mitigate the limited sample count and improve generalization, the Phase 1 training set was expanded fourfold via offline augmentation, while the validation and testing sets remained unmodified to preserve the integrity of the evaluation. The final distribution of images is detailed in Table 2.

**Fig 4:**
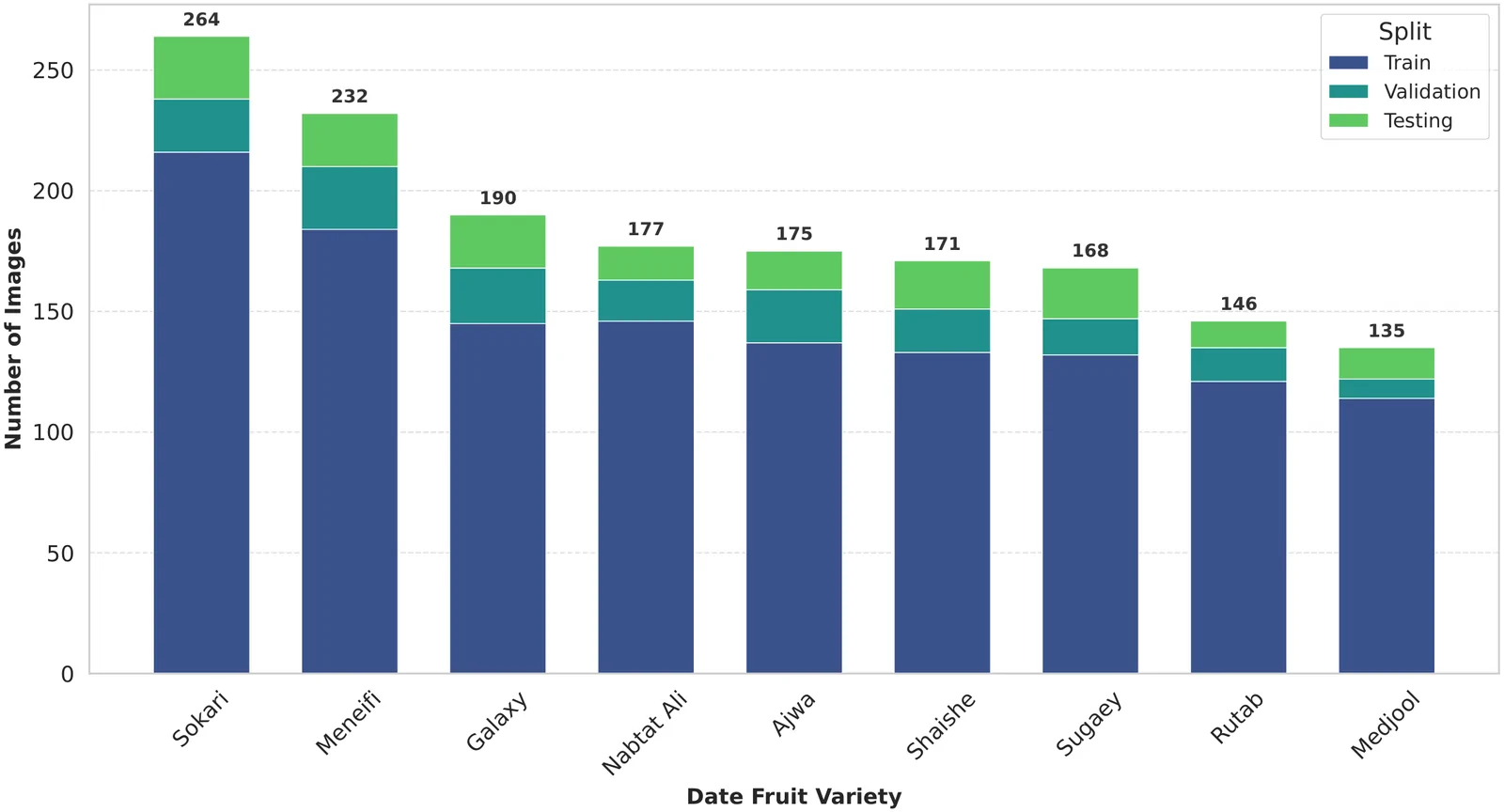
Class distribution. Number of images per variety in the training, validation, and test splits.

**Table 1:** Per-variety image counts. Number of original images for each of the nine date varieties. Counts are approximately preserved across the random training, validation, and testing splits.

Variety	Images	Variety	Images
Sokari	264	Shaishe	171
Meneifi	232	Sugaey	168
Galaxy	190	Rutab	146
Nabtat-Ali	177	Medjool	135
Ajwa	175	**Total**	**1658**

**Table 2:** Distribution of the Date Fruit Dataset. The training set was expanded via offline augmentation.

Dataset Split	Original Images	After Augmentation
Training Set	1328	5312
Validation Set	165	165
Testing Set	165	165
**Total**	**1658**	**5642**

### Preprocessing and augmentation strategy

A dual-stage augmentation framework was employed to address the specific requirements of each experimental phase.

#### Offline preprocessing (Phase 1).

Prior to the Single-Run Benchmark, the original training dataset (1,328 images) underwent offline augmentation to create a static, expanded dataset. Table 3 outlines the geometric and pixel-level transformations applied. This fixed training set was used for all models in Phase 1. Fig 5 demonstrates the visual impact of these transformations on a sample input.

**Table 3:** Offline preprocessing and augmentation configuration. These steps were applied prior to training to standardize inputs and enlarge the dataset size.

Hyperparameter	Configuration Details
Auto-Orient	Applied
Resize	Fit within 640×640 pixels
Flip	Horizontal, Vertical
Rotation	Range: $-15^{\circ }$ to $+15^{\circ }$
Shear	$\pm 10^{\circ }$ Horizontal, $\pm 10^{\circ }$ Vertical
Brightness	Range: ±15%
Exposure	Range: ±10%
Blur	Up to 2.5px
Noise	Up to 0.5% of pixels
Mosaic	Applied
Motion Blur	Length: 30px, Angle: $0^{\circ }$

**Fig 5:**
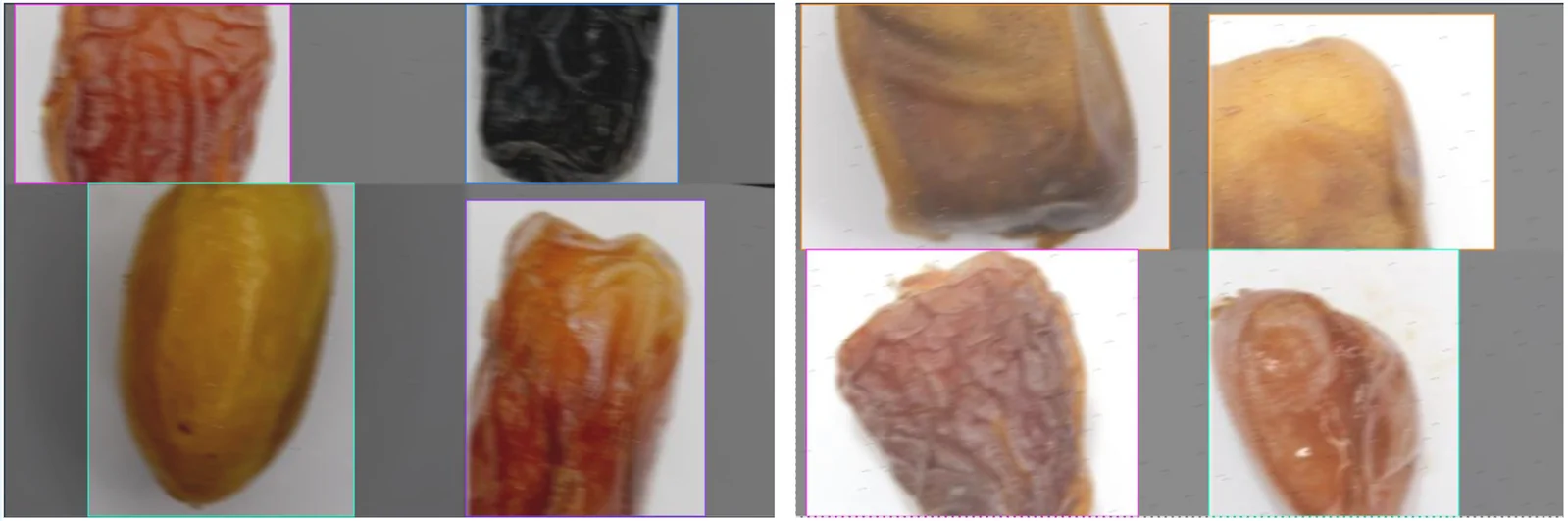
Sample of augmented images. Examples from the offline-augmented training set.

#### Online augmentation (Phase 2).

During the 5-fold cross-validation, offline augmentation was disabled and online augmentation was applied within the training loop to reduce overfitting. For the Ultralytics-trained models, Table 4 lists the augmentation hyperparameters, including Mosaic augmentation and Hue, Saturation, Value (HSV) color space perturbations. D-Fine-Nano used the native pipeline of its official implementation, consisting of random photometric distortion, zoom-out, cropping based on intersection over union (IoU), and horizontal flipping.

**Table 4:** Online augmentation hyperparameters. Used for the Ultralytics-trained models during the 5-fold cross-validation.

Hyperparameter	Configuration Details
Mosaic Augmentation	1.0 (Enabled)
Close Mosaic	Last 10 Epochs
Degrees (Rotation)	$\pm 0.0^{\circ }$ (Default)
Scale	0.5 (Default)
Flip Left-Right	0.5 (Default)
HSV-H (Hue)	0.015
HSV-S (Saturation)	0.7
HSV-V (Value)	0.4

### Cross-validation protocol

The 5-fold cross-validation (Phase 2) was designed to guard against data leakage and reduce dependence on a single train–test split. Folds were generated from the pool of 1,658 original, un-augmented images using grouped, stratified *k*-fold partitioning (*k* = 5, random seed 42; StratifiedGroupKFold in scikit-learn [26]). The two photographs of each same-fruit pair identified by the similarity audit share a group label, so no pair is split across folds, and each variety appears at approximately its dataset proportion in every fold. All 1,658 images were retained, and a single fold assignment was generated once and reused for all six models, ensuring that every architecture was trained and evaluated on identical folds.

In each of the five rounds, one fold (approximately 332 images) was held out for testing and the remaining four folds formed the outer training partition. From this outer training partition, 12.5% (approximately 165 images, also grouped and stratified) was set aside as an internal validation subset, leaving approximately 1,160 images for model training. The internal validation subset, not the held-out fold, was used to select the checkpoint (the epoch with the highest mAP@50–95) and the confidence threshold for precision, recall, and F1; the selected checkpoint and threshold were then applied once to the untouched held-out fold. Augmentation was applied only within the training data during each fold, so no augmented derivative of a held-out image could appear in the corresponding training partition.

### Lightweight object detection architectures

The study evaluates the “Nano” variants of five lightweight models, comparing the evolution of the CNN-based YOLO family against the emerging Transformer-based designs. The YOLO family has evolved through multiple architectural iterations, each balancing speed and accuracy for real-time applications [27]. These models are predominantly convolutional one-stage detectors designed for real-time applications. We evaluate four distinct iterations to trace recent architectural advancements: YOLOv8 [27,28], which adopted an anchor-free detection head and the C2f module; YOLOv10 [29], which notably eliminated Non-Maximum Suppression (NMS) via consistent dual assignments; YOLOv11 [30,31], which integrated the C3k2 block for improved feature integration; and the recently proposed YOLOv12 [32], which incorporates attention-centric modules while retaining convolutional downsampling layers and a convolutional detection head. The four YOLO variants are analyzed together as the CNN-based group. These are benchmarked against two Transformer-based architectures: D-Fine-Nano [33], a DEtection TRansformer (DETR)-based architecture that redefines the regression task using Fine-grained Distribution Refinement (FDR), and RT-DETR-Large [34]. RT-DETR-Large is deliberately included as a fixed, high-capacity accuracy reference—operating at more than an order of magnitude more computation than the Nano models—rather than as a lightweight competitor; accordingly, it is reported separately from the five lightweight detectors in the statistical comparison.

### Experimental environment

Standardization was supported by running all experiments in Google Colab Pro+ with the fixed software versions listed in Table 5. A dual-tier hardware strategy was adopted to characterize model behavior under both training and deployment-oriented inference conditions. High-throughput training was performed on an NVIDIA A100 GPU, and inference latency was benchmarked on both the A100 and NVIDIA T4 GPUs. The NVIDIA T4 was selected as the deployment-oriented platform because it is a low-profile PCIe inference accelerator that can be integrated into tethered industrial-PC-based vision systems [35].

**Table 5:** Hardware and software environment specifications. Hardware and software used for training and inference benchmarking. The lower block lists the environment of the grouped 5-fold cross-validation (Phase 2).

Component	Specification
Primary GPU (Phase 1 Training and Inference)	NVIDIA A100-SXM4 (40 GB VRAM)
Secondary GPU (Inference / Deployment Benchmark)	NVIDIA Tesla T4 (16 GB VRAM)
CPU Processor	Intel(R) Xeon(R) CPU @ 2.20GHz (2 vCPUs)
RAM	83.5 GB System RAM
Operating System	Ubuntu 22.04.3 LTS
Deep Learning Framework	PyTorch 2.5.1 + cu124
Detection Framework	Ultralytics 8.4.84 (YOLO & RT-DETR)
Python Version	3.11.11
CUDA Version	12.4
**Grouped cross-validation (Phase 2)**	
GPU	NVIDIA A100-SXM4 (80 GB VRAM)
Deep Learning Framework	PyTorch 2.11.0 + cu128
Detection Framework	Ultralytics 8.4.84 (YOLO & RT-DETR)
D-Fine Implementation	Official repository, commit 956d170
Python Version	3.13.15
CUDA Version	12.8

Regarding the software stack, all four YOLO variants (v8, v10, v11, and v12) and the RT-DETR-Large reference were trained within a single, unified release of the Ultralytics framework [28], which now provides native support for each of these architectures. Using a single framework version provided a consistent data-loading and augmentation pipeline across the four YOLO variants, reducing framework-level differences in their comparison. The D-Fine-Nano model was trained using its official implementation [33] to maintain fidelity to its specific fine-grained distribution refinement mechanisms. Table 5 details the hardware and software specifications, with the grouped cross-validation (Phase 2) environment listed separately.

### Training configuration

Training followed the two-phase design. In Phase 1, all models were trained on the offline-augmented dataset without online augmentation, and the Ultralytics models used early stopping with a patience of 15 epochs. In Phase 2, online augmentation was enabled for all models, as described in the Online augmentation section. All models were initialized with weights pre-trained on the COCO dataset and fine-tuned on the date fruit dataset to accelerate convergence. To support reproducibility, model training used a fixed random seed of 0, and the grouped cross-validation folds were generated with random seed 42.

Optimization settings were chosen per architecture rather than imposing identical hyperparameters across all models. The CNN-based YOLO models used stochastic gradient descent (SGD) with an initial learning rate of $1\times 10^{-2}$, the default initial learning rate in Ultralytics. The Transformer-based D-Fine and RT-DETR models used the AdamW optimizer [33,34] with base learning rates of $5\times 10^{-5}$ and $1\times 10^{-4}$, respectively; D-Fine’s backbone parameter groups retained the $4\times 10^{-4}$ rate of its official Nano configuration. Applying a single optimizer and learning rate across these different implementations would not reflect their respective training conventions. This study therefore benchmarks representative implementations using architecture-appropriate settings rather than performing an ablation in which all training factors are held constant. RT-DETR-Large was trained with a batch size of 16 because of the higher memory footprint of its large backbone. Table 6 summarizes the training hyperparameters.

**Table 6:** Training hyperparameter configuration. Settings for the CNN and Transformer-based models. For the YOLO models, the batch size was 64 in the Phase 1 single-run benchmark and 128 in the Phase 2 cross-validation.

Hyperparameter	YOLO Models	D-Fine-Nano	RT-DETR-Large
Initialization	COCO Pre-trained	COCO Pre-trained	COCO Pre-trained
Batch Size	64 / 128	16	16
Input Resolution	640×640	640×640	640×640
Optimizer	SGD	AdamW	AdamW
Initial Learning Rate	$1\times 10^{-2}$	$5\times 10^{-5}$ (backbone $4\times 10^{-4}$)	$1\times 10^{-4}$
Momentum	0.937	—	—
Weight Decay	$5\times 10^{-4}$	$1\times 10^{-4}$	$1\times 10^{-4}$
Single-Run Epochs	100 (patience 15)	100	100 (patience 15)
5-Fold Epochs	100	100	100
Learning Rate Schedule	Linear Decay	MultiStepLR	Linear Decay
Warm-up Period	3 Epochs	250 Iterations	3 Epochs

### Evaluation metrics

Model performance was quantified using standard metrics established by the COCO challenge [36] and specific latency constraints relevant to real-time sorting deployment.

#### Detection accuracy.

The primary accuracy metric is mean average precision (mAP), the mean of the per-class average precision (AP), where AP is the area under the precision–recall curve [37]. We report mAP@50, evaluated at an IoU threshold of 0.5, and mAP@50–95, averaged over IoU thresholds from 0.50 to 0.95. For all six models and in both experimental phases, detections produced by each implementation’s standard post-processing (Ultralytics validation at a confidence threshold of 0.001; D-Fine’s official post-processor) were exported and scored using the COCO evaluation API (pycocotools) [36].

Precision and recall were computed separately for each variety at an IoU threshold of 0.5 as


$$\begin{array}{c} P_{c}=\frac{{TP}_{c}}{{TP}_{c}+{FP}_{c}},\;R_{c}=\frac{{TP}_{c}}{{TP}_{c}+{FN}_{c}}, \end{array}$$ (1)

where ${TP}_{c}$, ${FP}_{c}$, and ${FN}_{c}$ denote the true positives, false positives, and false negatives for variety *c*. Precision and recall were then macro-averaged across the nine varieties, and the reported F1-score is the harmonic mean of these averaged values:


$$\begin{array}{c} F1=\frac{2PR}{P+R}, \end{array}$$ (2)

where *P* and *R* denote the macro-averaged precision and recall.

Precision, recall, and F1 were computed at the confidence threshold that maximized F1 on a validation subset not used for final reporting (the Phase 1 validation subset in Phase 1 and the internal validation subset in Phase 2), and this threshold was then applied unchanged to the corresponding test data. An operational system would instead tune the threshold to the false-positive tolerance of the sorting line.

#### Inference latency and efficiency.

We adopt 30 FPS, a common frame rate for conveyor inspection cameras [17], as the real-time threshold, corresponding to a maximum per-frame latency of 33.3 ms. Latency was measured on the Phase 1 test images at batch size 1, reflecting single-stream conveyor inspection, after 200 warm-up iterations, and is reported as the median over the test set. On the T4, models were evaluated in FP16 to leverage Tensor Core acceleration when supported; D-Fine-Nano was measured in full precision (FP32).

The reported model-side end-to-end latency is the sum of three stages measured using each framework’s own instrumentation: preprocessing (resize/letterbox, normalization, and host-to-device transfer), inference (the forward pass), and post-processing (NMS and box decoding). These measurements cover detector computation only and exclude image acquisition, camera triggering, frame transfer, decision logic, actuator communication, and physical ejection; they therefore do not represent end-to-end sorting-line latency or throughput. Computational complexity is additionally reported as FLOPs and model size at an input resolution of 640×640.

### Deployment optimization via model export

Deployment behavior beyond PyTorch inference was characterized by exporting the four YOLO models with the Ultralytics model.export API to ONNX [38] in FP32 and to TensorRT [39] engines in FP16, both at an input resolution of 640×640. ONNX is an open model-interchange format supported by multiple inference runtimes, whereas TensorRT is NVIDIA’s inference optimizer, which applies layer fusion, kernel auto-tuning, and reduced-precision execution on NVIDIA GPUs. The exported models were benchmarked on the T4 using the timing protocol described above.

The effect of FP16 conversion on accuracy was quantified by evaluating each Phase 1 YOLO checkpoint and its corresponding TensorRT FP16 engine on the Phase 1 test set on the T4 using an identical Ultralytics validation protocol. D-Fine-Nano and RT-DETR-Large were not included in the export benchmark because a unified FP16 export path across their deployment ecosystems was not available within our pipeline. This was a scoping decision and should not be interpreted as evidence that these detectors are less deployable.

## Results

Results are reported for the five lightweight detectors, with RT-DETR-Large retained as a high-capacity reference. Phase 1 results for detection accuracy, inference latency, and ONNX and TensorRT export are presented first, followed by the Phase 2 cross-validation results and statistical comparison.

### Detection performance (Phase 1)

Table 7 summarizes the Phase 1 single-run results. On this controlled dataset, all architectures achieved high accuracy within a narrow range (mAP@50–95 between 98.8% and 100.0%), as illustrated in Fig 6. D-Fine-Nano and the RT-DETR-Large reference reached the highest mAP@50–95 (100.0%), while the four CNN Nano variants were closely grouped. These near-ceiling values should be interpreted in light of the single-object protocol, in which each image contains one large, centered fruit against a clean background; they should not be taken as evidence of multi-object detection capability under conveyor conditions. Five of the 165 test images were identified by the similarity audit as repeat photographs of fruits that also appear in the Phase 1 training subset; excluding these images changed mAP@50–95 by at most 0.1 percentage points for any model. Representative detections are shown in Fig 7.

**Table 7:** Phase 1 single-run benchmarking results. Accuracy and model-side end-to-end latency on the 165-image test set. Best results are bolded.

Model	mAP	mAP	Precision	Recall	F1	Latency (ms)		FLOPs	Params	Size (MB)
	@50–95	@50				A100	T4			
YOLOv8-Nano	98.8	99.1	95.0	94.4	94.7	**9.4**	**9.3**	8.1G	3.01M	6.25
YOLOv10-Nano	99.8	**100.0**	**100.0**	97.4	98.7	10.4	10.2	6.5G	**2.27M**	5.76
YOLOv11-Nano	99.2	99.5	95.7	97.9	96.8	11.9	14.5	6.3G	2.58M	**5.47**
YOLOv12-Nano	99.3	99.5	98.3	98.1	98.2	17.2	17.5	6.3G	2.56M	5.52
D-Fine-Nano	**100.0**	**100.0**	99.5	**100.0**	99.8	30.4	28.9	**3.5G**	3.73M	14.3
RT-DETR-Large	**100.0**	**100.0**	**100.0**	**100.0**	**100.0**	43.4	45.0	103.5G	32.0M	66.25

**Fig 6:**
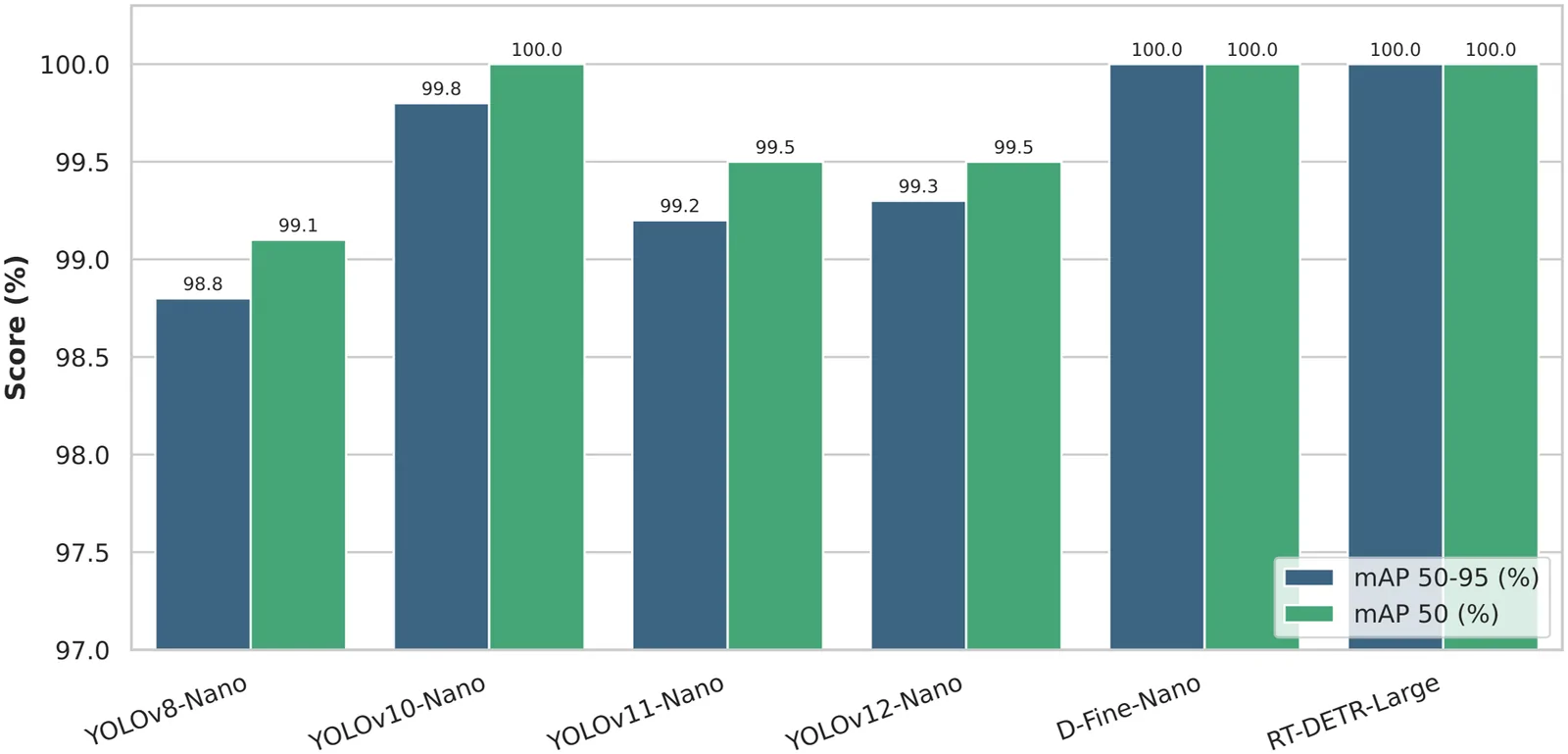
Comparison of detection accuracy. Phase 1 mAP@50–95 and mAP@50 for each model.

**Fig 7:**
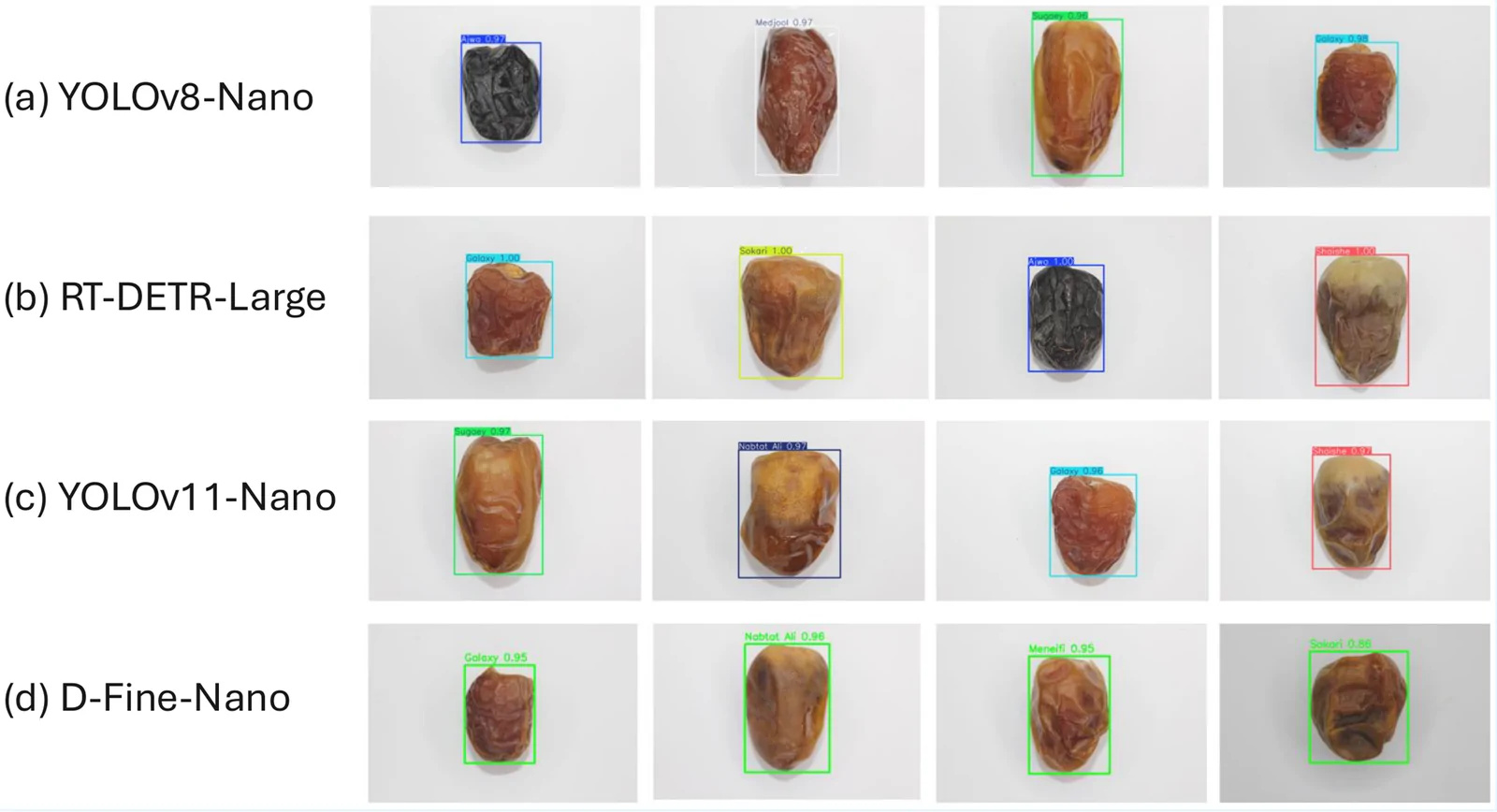
Detection samples. Predictions of representative CNN and Transformer detectors: a) YOLOv8-Nano, b) RT-DETR-Large, c) YOLOv11-Nano, and d) D-Fine-Nano.

Fig 8 shows the confusion matrices of the representative detectors. D-Fine-Nano (Fig 8d) showed near-perfect diagonal concentration across the nine varieties, with no apparent inter-variety misclassifications. YOLOv8-Nano (Fig 8a) also showed strong diagonal concentration, with most residual errors involving the Nabtat-Ali class. It produced six false-positive Nabtat-Ali detections on background regions and labeled five Sokari fruits as Nabtat-Ali. The former lowers Nabtat-Ali precision, whereas the latter reduces Sokari recall, consistent with the per-class metrics in Table 8. The remaining varieties were detected with few or no errors.

**Fig 8:**
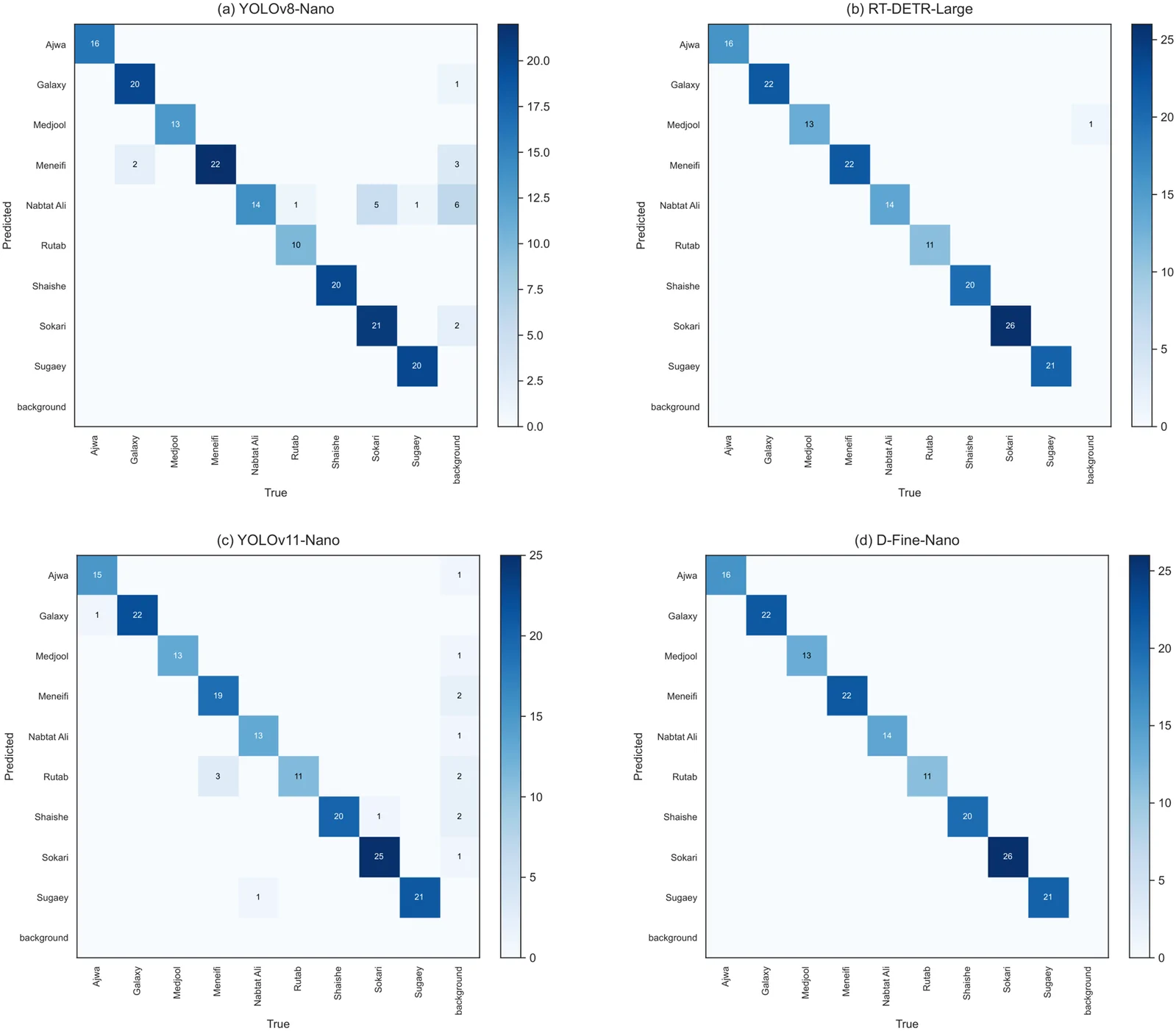
Confusion matrices of representative CNN and Transformer detectors. a) YOLOv8-Nano, b) RT-DETR-Large, c) YOLOv11-Nano, and d) D-Fine-Nano. Matrices are computed at the default confidence threshold of 0.25; the per-class precision and recall in Table 8 use the F1-optimal threshold selected on the validation subset, which accounts for small numeric differences from the raw matrix counts.

**Table 8:** Per-class performance of YOLOv8-Nano on the test set. Precision, recall, and F1-score for each date variety (165 test images), at the confidence threshold selected on the Phase 1 validation subset. F1 is computed as the harmonic mean of precision and recall.

Variety	Precision	Recall	F1-score	Test images
Ajwa	1.000	1.000	1.000	16
Galaxy	1.000	0.864	0.927	22
Medjool	1.000	1.000	1.000	13
Meneifi	0.917	1.000	0.957	22
Nabtat-Ali	0.636	1.000	0.778	14
Rutab	1.000	0.909	0.952	11
Shaishe	1.000	1.000	1.000	20
Sokari	1.000	0.769	0.870	26
Sugaey	1.000	0.952	0.976	21

Table 8 summarizes the per-variety performance of YOLOv8-Nano. F1 was at least 0.97 for Ajwa, Medjool, Shaishe, and Sugaey, and the main errors involved Nabtat-Ali, Sokari, and Galaxy. Nabtat-Ali precision was 0.64 because of false-positive predictions on background and on some Sokari fruits. Sokari recall was 0.77, mainly because several Sokari fruits were classified as Nabtat-Ali, and Galaxy recall was 0.86. The other CNN detectors showed different class-specific weaknesses; for example, YOLOv11-Nano had lower recall for Nabtat-Ali (0.86) and lower precision for Rutab (0.73). For D-Fine-Nano and RT-DETR-Large, precision, recall, and F1 were at least 0.95 for every variety.

The Sokari–Nabtat-Ali confusion may reflect similarities in color and surface texture or within-class variation, although the available images do not support attributing it to a single visual characteristic. Class frequency alone does not explain the errors. Sokari is the largest class, with 264 images, but has one of the lowest F1 scores, whereas Medjool is the smallest class, with 135 images, and reaches an F1 of 1.000. Maturity-stage variation may also affect appearance, but the dataset does not provide maturity labels.

### Inference latency and hardware scaling

As shown in Table 7, all five lightweight architectures operated below the 33.3 ms model-side latency threshold corresponding to 30 FPS on both GPUs. The high-capacity RT-DETR-Large reference did not meet this requirement, recording 45.0 ms on the T4.

Fig 9 illustrates the effect of hardware tier on model-side latency. For the CNN Nano models, the difference between the A100 and T4 was small, at most a few milliseconds, and for the smallest models the two GPUs were effectively indistinguishable. The four CNN Nano variants achieved the lowest latencies, 9.3–17.5 ms on the T4, with YOLOv8-Nano the fastest. D-Fine-Nano recorded 28.9 ms on the T4, roughly three times the fastest CNN but still within the 30 FPS model-side threshold. We note that D-Fine was timed in FP32 because stable FP16 inference was not available for D-Fine-Nano in our setup, and that its preprocessing used an unoptimized image-resize path; its end-to-end latency is therefore a conservative estimate. Residual run-to-run variation on the shared cloud GPUs was on the order of a few milliseconds, so small latency differences between similarly sized models should not be over-interpreted.

**Fig 9:**
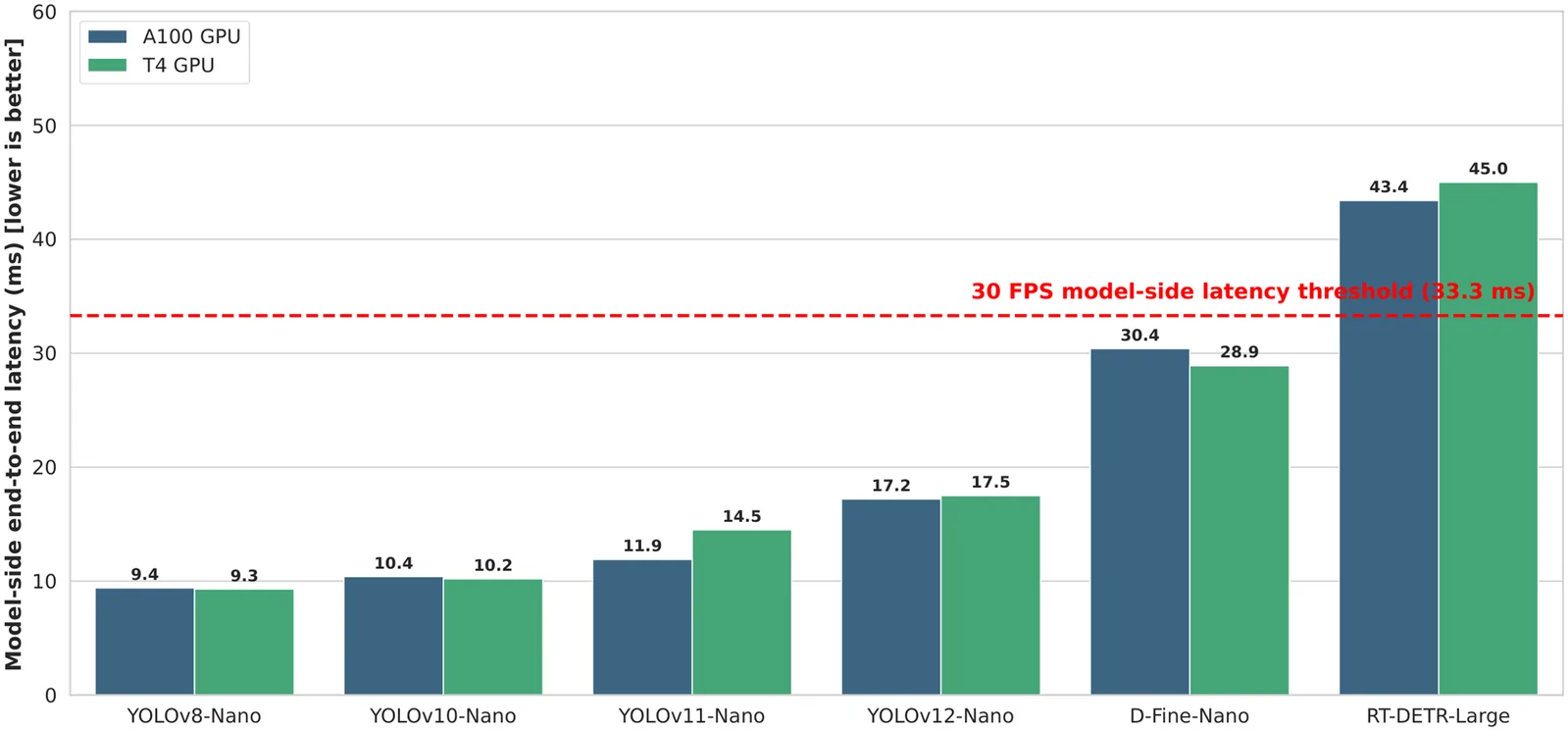
Impact of hardware acceleration. Model-side end-to-end latency (preprocessing + inference + post-processing) on the A100 and T4.

### Speed–accuracy trade-off

The model-level speed–accuracy trade-off is illustrated in Fig 10. The green shaded region represents the model-side latency region corresponding to 30 FPS (latency < 33.3 ms). All five lightweight models fall within this model-side 30 FPS region at high accuracy, with the four CNN Nano variants clustered in the low-latency region and D-Fine-Nano positioned at slightly higher latency but comparable accuracy. RT-DETR-Large matches the highest accuracy but falls outside the latency boundary, and therefore does not meet the 30 FPS model-side latency threshold on this hardware.

**Fig 10:**
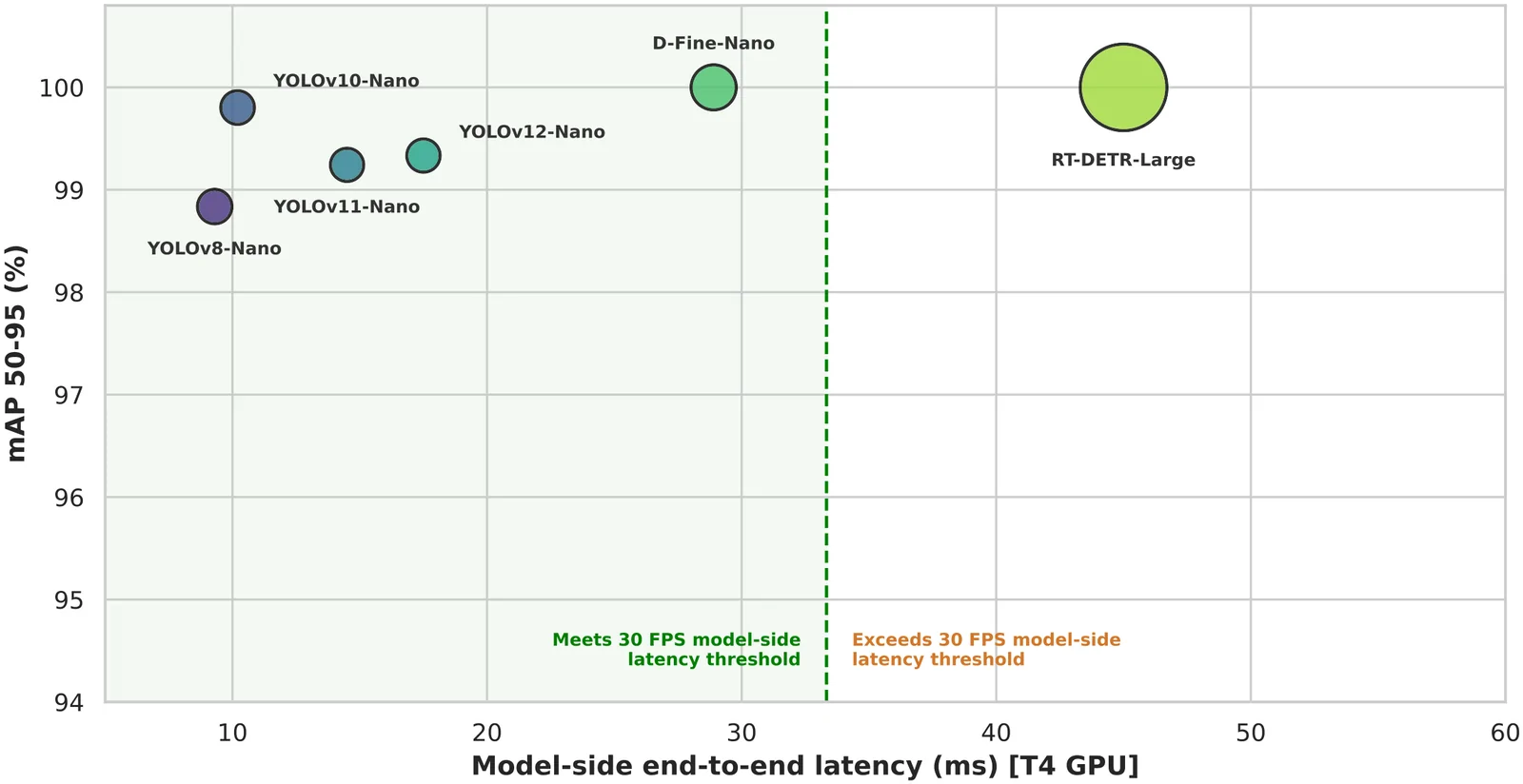
Speed–accuracy trade-off analysis. Measured on the T4. Latency values are model-side end-to-end (preprocessing + inference + post-processing). The circle size reflects the model size.

### Deployment optimization results

The four YOLO Nano models were exported to ONNX and TensorRT FP16 as described in the Materials and Methods section. D-Fine-Nano and RT-DETR-Large were not included because a unified FP16 export path was not available within our pipeline, so these results characterize the YOLO models only and do not indicate that Transformer detectors are less deployable. Table 9 reports ONNX and TensorRT inference latency on the NVIDIA T4 GPU, TensorRT end-to-end latency, and the change in mAP@50–95 after TensorRT FP16 conversion.

**Table 9:** TensorRT export performance on the NVIDIA T4 GPU. Median Phase 1 test-set latency, ONNX-to-TensorRT inference speedup, and FP16 change in mAP@50–95.

Model	ONNX	TensorRT	TensorRT	Speedup	ΔmAP
	Inference (ms)	Inference (ms)	End-to-end (ms)	(×)	(pp)
YOLOv8-Nano	5.06	**3.31**	7.33	1.53	−1.2
YOLOv10-Nano	9.02	3.52	**6.27**	2.56	+0.2
YOLOv11-Nano	9.91	3.62	8.06	**2.74**	+0.7
YOLOv12-Nano	12.70	4.97	9.88	2.56	+0.4

TensorRT FP16 reduced the inference-stage latency of all four YOLO models to 3.31–4.97 ms, corresponding to 1.53–2.74× speedups relative to ONNX inference. YOLOv8-Nano had the lowest inference-stage latency at 3.31 ms, followed closely by YOLOv10-Nano at 3.52 ms. Because this difference is small relative to the observed run-to-run variation, the ordering should be interpreted as specific to the tested configuration. YOLOv10-Nano achieved the lowest model-side end-to-end latency at 6.27 ms. With inference accelerated, preprocessing and post-processing accounted for 44–55% of the TensorRT end-to-end latency, limiting the end-to-end gains achievable through inference-stage optimization alone.

TensorRT FP16 conversion reduced YOLOv8-Nano mAP@50–95 by 1.2 percentage points on the Phase 1 test set, whereas YOLOv10-, YOLOv11-, and YOLOv12-Nano showed no observed accuracy degradation. The small positive changes for these three models were treated as numerical variation between the PyTorch and TensorRT evaluations rather than evidence of accuracy gains. FP16 export therefore preserved accuracy for three of the four models.

### Cross-validation results (Phase 2)

The grouped 5-fold cross-validation results are summarized in Table 10, with complete fold-wise metrics reported in Table 11. Mean mAP@50–95 among the five lightweight models ranged from 0.9963 to 0.9989. YOLOv12-Nano had the numerically highest mean at 0.9989 and the lowest fold-to-fold standard deviation at 0.0014. The RT-DETR-Large reference achieved a mean of 0.9937 with a standard deviation of 0.0039. The differences in mean mAP@50–95 among the lightweight models were of the same order as their fold-to-fold variation. The fold-wise distributions and mean model ranks are shown in Fig 11.

**Table 10:** Grouped 5-fold cross-validation performance (mAP@50–95). Mean, standard deviation (SD), and 95% confidence interval (CI) across five folds. *RT-DETR-Large is a high-capacity reference and is excluded from the significance test.

Model Architecture	Mean mAP@50–95	SD	95% CI
YOLOv12-Nano	0.9989	0.0014	[0.9971, 1.0000]
YOLOv11-Nano	0.9967	0.0042	[0.9914, 1.0000]
YOLOv10-Nano	0.9966	0.0038	[0.9919, 1.0000]
D-Fine-Nano	0.9964	0.0026	[0.9932, 0.9997]
YOLOv8-Nano	0.9963	0.0025	[0.9932, 0.9994]
RT-DETR-Large${\;}^{*}$	0.9937	0.0039	[0.9889, 0.9985]

**Table 11:** Fold-wise grouped 5-fold cross-validation results. Per-fold values with mean, SD, and 95% CI for mAP@50–95, mAP@50, precision, recall, and F1. *RT-DETR-Large is a high-capacity reference and is excluded from the significance test.

Model	Metric	Fold 1	Fold 2	Fold 3	Fold 4	Fold 5	Mean	SD	95% CI
**YOLOv8-Nano**	mAP@50–95	0.9995	0.9981	0.9934	0.9947	0.9957	0.9963	0.0025	[0.9932, 0.9994]
	mAP@50	0.9995	0.9981	0.9934	0.9947	0.9957	0.9963	0.0025	[0.9932, 0.9994]
	Precision	0.9842	0.9674	0.9561	0.9777	0.9681	0.9707	0.0108	[0.9573, 0.9840]
	Recall	0.9976	0.9852	0.9713	0.9878	0.9885	0.9861	0.0095	[0.9743, 0.9979]
	F1	0.9908	0.9762	0.9636	0.9827	0.9782	0.9783	0.0100	[0.9660, 0.9907]
**YOLOv10-Nano**	mAP@50–95	0.9995	0.9985	0.9900	0.9969	0.9979	0.9966	0.0038	[0.9919, 1.0000]
	mAP@50	0.9999	0.9989	0.9915	0.9969	0.9979	0.9970	0.0033	[0.9929, 1.0000]
	Precision	0.9857	0.9872	0.9670	0.9555	0.9777	0.9746	0.0134	[0.9580, 0.9912]
	Recall	0.9960	0.9806	0.9299	0.9751	0.9760	0.9715	0.0247	[0.9408, 1.0000]
	F1	0.9908	0.9839	0.9481	0.9652	0.9769	0.9730	0.0168	[0.9521, 0.9939]
**YOLOv11-Nano**	mAP@50–95	0.9967	1.0000	0.9894	0.9992	0.9983	0.9967	0.0042	[0.9914, 1.0000]
	mAP@50	0.9967	1.0000	0.9913	0.9997	0.9983	0.9972	0.0036	[0.9928, 1.0000]
	Precision	0.9900	1.0000	0.9706	0.9819	0.9813	0.9848	0.0110	[0.9712, 0.9984]
	Recall	0.9824	0.9828	0.9767	0.9936	0.9929	0.9857	0.0073	[0.9766, 0.9948]
	F1	0.9862	0.9913	0.9736	0.9877	0.9871	0.9852	0.0067	[0.9768, 0.9936]
**YOLOv12-Nano**	mAP@50–95	0.9998	1.0000	0.9967	0.9998	0.9982	0.9989	0.0014	[0.9971, 1.0000]
	mAP@50	0.9998	1.0000	0.9976	0.9998	0.9982	0.9991	0.0011	[0.9977, 1.0000]
	Precision	0.9906	1.0000	0.9871	0.9871	0.9511	0.9832	0.0187	[0.9600, 1.0000]
	Recall	0.9856	0.9967	0.9774	0.9937	0.9874	0.9881	0.0075	[0.9788, 0.9975]
	F1	0.9881	0.9984	0.9822	0.9904	0.9689	0.9856	0.0110	[0.9720, 0.9992]
**D-Fine-Nano**	mAP@50–95	0.9998	0.9955	0.9935	0.9949	0.9985	0.9964	0.0026	[0.9932, 0.9997]
	mAP@50	0.9998	0.9955	0.9939	0.9949	0.9985	0.9965	0.0025	[0.9934, 0.9996]
	Precision	0.9909	0.9613	0.9867	0.9763	0.9665	0.9763	0.0127	[0.9606, 0.9921]
	Recall	0.9870	0.9790	0.9634	0.9849	0.9799	0.9788	0.0093	[0.9673, 0.9903]
	F1	0.9889	0.9701	0.9749	0.9806	0.9732	0.9775	0.0074	[0.9683, 0.9868]
**RT-DETR-Large** ${\;}^{*}$	mAP@50–95	0.9904	0.9914	0.9915	0.9953	0.9997	0.9937	0.0039	[0.9889, 0.9985]
	mAP@50	0.9904	0.9914	0.9930	0.9953	0.9997	0.9939	0.0037	[0.9893, 0.9986]
	Precision	0.9656	0.9800	0.9565	0.9836	0.9873	0.9746	0.0130	[0.9584, 0.9908]
	Recall	0.9968	0.9975	0.9837	0.9937	0.9923	0.9928	0.0055	[0.9859, 0.9997]
	F1	0.9809	0.9887	0.9699	0.9886	0.9898	0.9836	0.0084	[0.9731, 0.9940]

**Fig 11:**
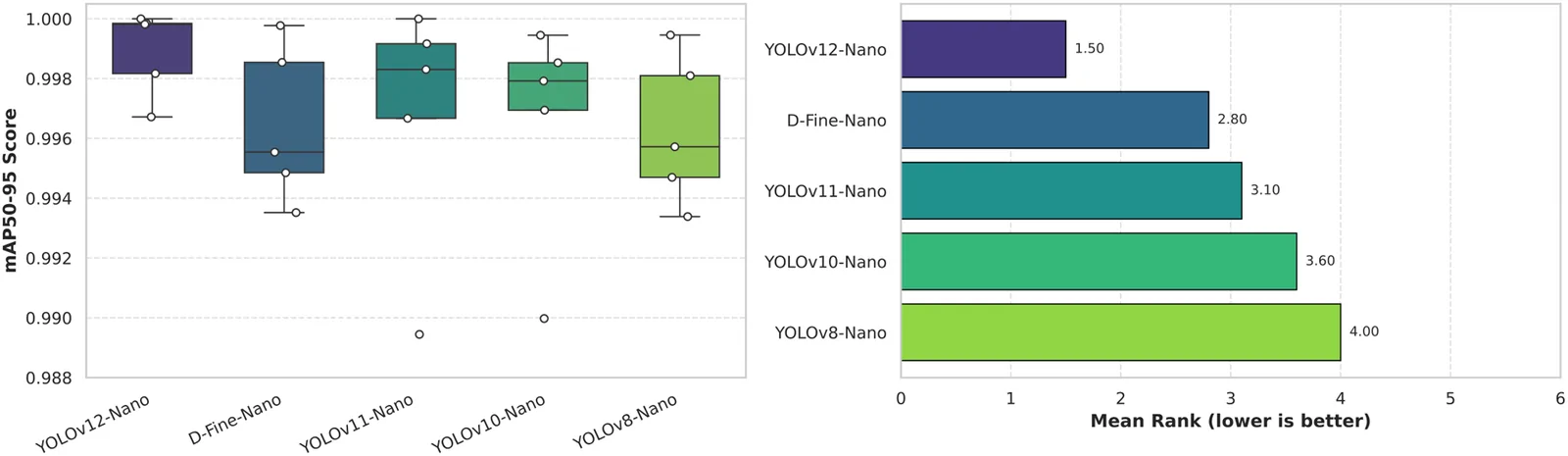
Distribution of cross-validation performance and model ranks. Left: mAP@50–95 across the five grouped folds. Right: mean ranks used in the Friedman analysis, where a lower rank indicates better performance.

Statistical comparison was restricted to the five lightweight models, with RT-DETR-Large retained as a high-capacity reference. The Friedman test [40], computed from the unrounded fold-level mAP@50–95 values, detected no statistically significant difference among the lightweight models ($\chi^{2}(4)=7.394$, *p* = 0.116). YOLOv12-Nano had the best mean rank of 1.5 and YOLOv8-Nano the worst at 4.0. However, this rank difference was smaller than the Nemenyi critical difference of 2.728, and no pairwise comparison was statistically significant at *p* < 0.05, as shown in Fig 12. Because the analysis is based on only five folds, its statistical power is limited, and this result does not establish that the models are equivalent. Accuracy alone therefore does not distinguish the lightweight detectors on this dataset; latency, fold-to-fold stability, model size, and deployment behavior provide a clearer basis for choosing between them.

**Fig 12:**
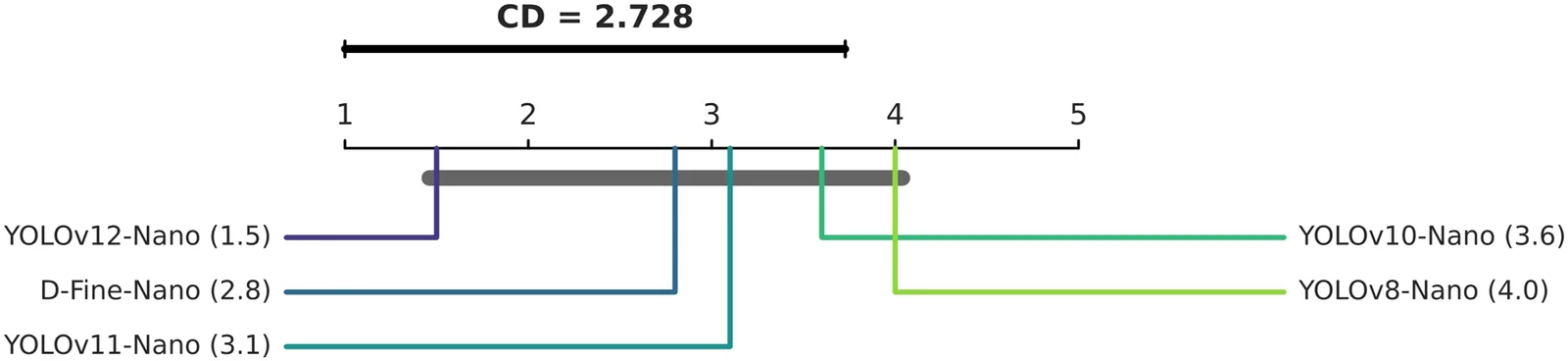
Critical Difference diagram. Mean ranks of the five lightweight models based on fold-level mAP@50–95. Models connected by a horizontal bar are not significantly different according to the Nemenyi post-hoc analysis (*p* > 0.05).

## Discussion

This model-level benchmark of controlled, single-fruit date-variety recognition highlights four practical considerations for deployment: measured latency did not track theoretical complexity, no statistically significant accuracy difference was detected among the lightweight models, fold-to-fold stability differed numerically across models, and TensorRT conversion affected the exported YOLO models differently.

### Theoretical complexity versus measured latency

YOLOv12-Nano illustrates the gap between theoretical computational cost and measured runtime. Although its 6.3 GFLOPs tied with YOLOv11-Nano for the lowest among the CNN variants, it had the highest model-side end-to-end latency of the four CNN models in every tested configuration (Tables 7 and 9). This indicates that FLOPs alone are insufficient to predict inference latency on a specific hardware and software stack, where implementation details and runtime behavior also contribute. Because low-level kernel, memory-access, and operator profiling were not performed, the observed latency differences cannot be attributed to a specific architectural mechanism.

### CNN versus Transformer trade-offs for industrial sorting

D-Fine-Nano achieved a mean cross-validation mAP@50–95 of 0.9964, within the range of the four CNN-based YOLO models (0.9963–0.9989). The Friedman–Nemenyi analysis detected no statistically significant accuracy difference among the lightweight models, so the results do not support an accuracy advantage for D-Fine-Nano or for the YOLO models. Under the tested T4 configurations, the YOLO models showed lower measured model-side latency than D-Fine-Nano; however, D-Fine-Nano was timed in FP32 whereas the YOLO models were timed in FP16, so this latency comparison should be interpreted as implementation-specific rather than as an inherent CNN–Transformer difference. D-Fine-Nano nonetheless met the 30 FPS model-side threshold on the T4 and remains a viable option where its higher latency is acceptable.

The export results also highlight that deployment behavior depends on the software and runtime pipeline, not only on model architecture. The tested YOLO models had a direct TensorRT FP16 deployment path, whereas comparable export behavior was not evaluated for D-Fine-Nano or RT-DETR-Large. Even within the YOLO family, FP16 conversion reduced YOLOv8-Nano mAP@50–95 by 1.2 percentage points without degrading the other three models, so the accuracy of each exported engine should be checked before deployment.

### Cross-validation stability

Fold-to-fold stability provides an additional view of how consistently each model performs across data partitions. YOLOv12-Nano showed the lowest fold-to-fold standard deviation in mAP@50–95 among the lightweight models at 0.0014, indicating the most consistent numerical performance across the five grouped folds. Its TensorRT end-to-end latency, however, was 9.88 ms compared with 6.27 ms for YOLOv10-Nano. Given the absence of a statistically significant accuracy difference, the lower variability of YOLOv12-Nano should be interpreted as a stability characteristic rather than a demonstrated performance advantage, particularly because the standard deviation is estimated from only five folds. Moreover, fold-to-fold stability on this controlled dataset does not establish batch-to-batch consistency on an operational sorting line, which would require evaluation across independent production batches and imaging conditions.

### Implications for the date fruit industry

The model-side latencies observed on the T4 indicate that lightweight detectors can meet the computational requirements of real-time single-fruit recognition under the controlled conditions evaluated here. This supports their potential use as the vision component of future date-sorting systems, particularly for SMEs, but does not establish economic feasibility.

A practical system could combine an industrial RGB camera, controlled illumination, a conveyor, and a tethered PC equipped with a T4-class GPU accelerator. The lightweight models evaluated here had substantially fewer parameters and lower measured T4 latency than the RT-DETR-Large reference, which may reduce the computing requirements of such a system. However, practical deployment would still depend on factors not evaluated in this study, including fruit singulation, image acquisition, actuator response, system integration, and hardware cost. Validation on a complete camera-to-ejector sorting line is therefore required before operational performance can be established.

### Limitations and future directions

The main limitation is the controlled nature of the dataset. Each image contains one large, centered fruit against a clean background under uniform illumination, making the task substantially easier than operational sorting. The near-ceiling mAP@50–95 values should therefore not be interpreted as evidence of robustness to occlusion, variable orientation, conveyor backgrounds, dust, mixed maturity stages, or multiple fruits. Because the source dataset lacks physical-fruit provenance metadata, same-fruit repeat photographs were identified through a similarity audit with visual inspection and kept within single cross-validation folds; datasets with physical-fruit identifiers would allow this grouping to be applied directly. Future evaluation should use independently collected conveyor-line images with greater visual and operational variability.

A second limitation is implementation comparability. The YOLO variants and RT-DETR-Large were trained within Ultralytics, whereas D-Fine-Nano used its official implementation. Although all models were scored with a common evaluator, the two frameworks differ in preprocessing, online augmentation, optimization, and timing instrumentation, and D-Fine-Nano was timed in FP32 on the T4, whereas the YOLO models were timed in FP16. Comparisons involving D-Fine-Nano should therefore be interpreted as comparisons of the tested implementations rather than of architectures in isolation. This concern is reduced for comparisons among the four YOLO variants, which shared the same framework and data pipeline.

Finally, the reported latency is model-side only and does not include image acquisition, camera triggering, decision logic, actuator communication, or physical ejection. The T4 also represents a tethered GPU accelerator rather than a low-power embedded platform. Future work should evaluate multi-fruit scenes, partially occluded fruits, images captured from a moving conveyor, and mixed-variety batches, measure complete camera-to-ejector latency and energy use, and benchmark lower-power hardware such as the NVIDIA Jetson Orin Nano.

## Conclusion

This study presented a model-level benchmark of lightweight CNN and Transformer detectors for controlled, single-fruit date-variety recognition, with model-side inference evaluated on an NVIDIA A100 and a T4-class edge GPU/industrial-PC accelerator. Under grouped 5-fold cross-validation, the five lightweight models reached mean mAP@50–95 values of 0.9963 to 0.9989, and a Friedman–Nemenyi analysis detected no statistically significant accuracy difference among them. All five met the 30 FPS threshold for model-side detection latency on the T4 under the tested conditions; image acquisition, camera triggering, actuator communication, and physical ejection were not measured and would add to full sorting-line latency. Model selection is therefore primarily an engineering decision based on latency, stability, and deployment behavior.

For the evaluated setup, namely the controlled 1,658-image, nine-variety single-fruit dataset, NVIDIA T4 hardware, and TensorRT FP16 export pipeline, YOLOv10-Nano represents one practical configuration for SME-oriented deployment, with the lowest end-to-end latency at 6.27 ms and no observed accuracy degradation after FP16 conversion. YOLOv8-Nano had the lowest inference-stage latency at 3.31 ms but showed a 1.2-percentage-point decrease in mAP@50–95 after conversion, whereas YOLOv12-Nano had the numerically highest cross-validation mean and the lowest fold-to-fold standard deviation at a higher end-to-end latency of 9.88 ms. These results reflect speed, stability, and deployment trade-offs rather than the overall superiority of any single model. Because the Transformer detectors were not included in the export benchmark, the export results apply to the tested YOLO pipeline and do not show that CNN detectors are generally more deployable.

Within this controlled setting, the evaluated Nano-scale detectors recognized date varieties with high accuracy while meeting the model-side latency budget on the tested T4-class accelerator. Any selected model should be revalidated on data from the target sorting line with the intended hardware and export configuration; with that step, this benchmark provides a foundation for moving date-variety recognition from controlled images toward operational sorting lines.

## Additional information

**Licensing and Commercial Use:** The object detection architectures benchmarked in this study are implemented via open-source libraries. The YOLO variants (v8, v10, v11, v12) and the RT-DETR reference were trained using the Ultralytics framework, which is distributed under the AGPL-3.0 license and alternative commercial licensing terms [41]; D-Fine was used through its official repository under its own open-source license. Users are responsible for ensuring that their intended deployment complies with the applicable license.

**Declaration of generative AI:** During the preparation of this work, the authors used Google Gemini, and Anthropic Claude to assist with language editing, readability, and structural refinement. After using these tools, the authors reviewed and edited the content as needed and take full responsibility for the content of the published article.
